# Targeting Angiogenesis for Controlling Neuroblastoma

**DOI:** 10.1155/2012/782020

**Published:** 2011-08-25

**Authors:** Subhasree Roy Choudhury, Surajit Karmakar, Naren L. Banik, Swapan K. Ray

**Affiliations:** ^1^Department of Pathology, Microbiology, and Immunology, University of South Carolina School of Medicine, Columbia, SC 29209, USA; ^2^Department of Neurosciences, Medical University of South Carolina, Charleston, SC 29425, USA

## Abstract

Neuroblastoma, a progressive solid tumor in childhood, continues to be a clinical challenge. It is highly vascular, heterogeneous, and extracranial tumor that originates from neural crest. Angiogenesis, genetic abnormalities, and oncogene amplification are mainly responsible for malignant phenotype of this tumor. Survivability of malignant neuroblastoma patients remains poor despite the use of traditional therapeutic strategies. Angiogenesis is a very common and necessary pre-requisite for tumor progression and metastasis. Angiogenesis is also a major factor in making malignant neuroblastoma. Thus, prevention of angiogenesis can be a highly significant strategy in the treatment of malignant neuroblastoma. Here, we summarize our current understanding of angiogenesis in malignant neuroblstoma and describe the use of experimental anti-angiogenic agents either alone or in combination therapy. This review will clearly indicate the importance of angiogenesis in the pathogenesis of malignant neuroblastoma, its prevention as a promising therapy in preclinical models of malignant neuroblastoma, and prospective clinical trials.

## 1. Introduction

**Figure 1:**
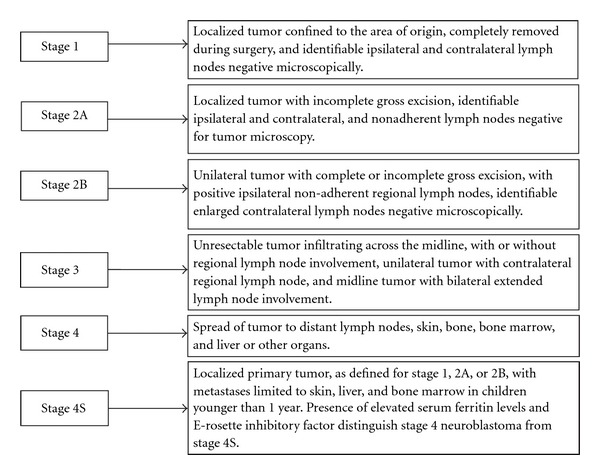
Staging system for neuroblastoma according to the INSS.

Neuroblastoma is the most common, extracranial, and heterogeneous solid tumor in children, and it accounts for approximately 15% of pediatric cancer deaths with an estimated incidence of 1 per 7000 births in the USA [1, 2]. This embryonal cancer of postganglionic sympathetic nervous system arises from adrenal gland and less frequently metastasizes in other places such as chest, neck, lymph nodes, pelvis, liver, and bone. The prognosis is variable and depends on several factors. Neuroblastoma is characterized by its intriguing clinical behaviors that include spontaneous differentiation and regression, maturation into benign ganglioneuroma, and deadly metastatic tumor. This childhood neoplasm is staged clinically according to the International Neuroblastoma Staging System (INSS) (Figure 1). The genetic features of neuroblastoma include *N-Myc* oncogene amplification or allelic loss, near triploid karyotype, deletion of short arm of chromosome 1, and high expression of neurotrophin receptors (TrkA and TrkB), all of which are associated with malignant transformation and progression of this disease. Multimodal treatment approaches including myeloablative chemotherapy, radionuclide therapy, immunotherapy, and apoptosis-inducing therapy are evaluated as traditional therapeutic strategies for controlling the malignant growth of the tumors. Despite aggressive conventional treatments and diagnosis techniques in neurosurgery, the survival rate for patients with neuroblastoma remains poor because the majority of children older than 1 year of age with advanced stage neuroblastoma die from progressive disease and only 40% of children over 4 years old with neuroblastoma survive for 5 years, emphasizing the urgent need for the development of innovative therapeutic strategies for treatment of malignant neuroblastoma. Malignant neuroblastoma is a highly vascularized solid tumor that requires access to blood vessels for growth, invasion, and metastasis [3]. Emerging treatments with the delivery of antiangiogenic molecules can thereby hinder neovascularization and arrest the spread of this pediatric tumor. Novel therapeutic approaches with the angiogenic inhibitors are expected to improve patient survivability by reducing morbidity, mortality, and drug-related toxicity.

## 2. Angiogenesis in Human Neuroblastoma

Angiogenesis is a process of development of intrinsic vascular network, and it is a prerequisite for progression and metastatic spread of solid tumors like neuroblastoma where new capillaries sprout from preexisting vessels and the transition from avascular to vascular phase occurs via neovascularization. Tumor angiogenesis is characterized by cascade of events involving mainly dissolution of vascular basal membrane, increased vascular permeability, and degradation of extracellular matrix resulting in endothelial cell migration, invasion, proliferation, and tube formation [4–7]. Finally, the recruitment of perivascular supporting cells such as pericytes, subsequent inhibition of endothelial proliferation, basement membrane reconstitution, and structural reorganization into a functional complex formation stabilize the microvasculature. 

Angiogenesis is mediated by multiple regulatory factors such as growth factors, adhesion molecules, and matrix degrading enzymes. Activation of endothelial cell proliferation and migration are mainly regulated by receptor tyrosine kinase ligands such as vascular endothelial growth factor (VEGF), fibroblast growth factor-2 (FGF-2), platelet derived growth factor (PDGF), epidermal growth factor (EGF), transforming growth factor-alpha (TGF-*α*), and angiopoietins (Ang-1 and Ang-2). The naturally occurring endogenous angiogenesis inhibitors that affect neuroblastoma growth *in vivo* includes angiostatin, endostatin, tumstatin, canstatin, tissue inhibitors of matrix metalloproteinases (MMP), and so forth. An angiogenic switch actually maintains the balance between angiogenic activators and inhibitors and maintains the endothelial cells in an angiogenic or quiescent stage. Malignant growth of human neuroblastoma is highly dependent on angiogenesis. Therefore, anti-angiogenic strategies can be effective in inhibiting tumor cell dissemination and metastasis in highly vascular neuroblastoma [3–7].

## 3. Angiogenesis Stimulatory Factors in Human Neuroblastoma

### 3.1. Angiogenic Growth Factors and Their Implications

#### 3.1.1. VEGF and VEGFR Family

VEGF (46 kDa) is an endothelial specific mitogen that plays a crucial role in pathogenesis and neovascularization of neuroblastoma. VEGF signaling plays a regulatory role in neuroblastoma angiogenesis via a paracrine mechanism through two specific tyrosine kinase VEGF receptors: VEGFR-1 (or Flt-1) and VEGFR-2 (or KDR) at the surface of the endothelial cells. The most potent angiogenic factor to promote endothelial cell proliferation is VEGF-A. Encoded by a single gene, VEGF-A has several isoforms such as VEGF-A_121_, VEGF-A_165_, VEGF-A_189_, and VEGF-A_206_, which are generated by alternative exon splicing. The other members of the VEGF family include VEGF-B, VEGF-C, VEGF-D, and VEGF-E [8, 9]. VEGFR-2 is the major mediator of VEGF-induced endothelial cell proliferation, migration, and survival, and it acts as a potent microvascular permeability enhancer of VEGF. VEGFR-3/Flt-4 is a member of the same tyrosine kinase receptors of VEGF-C and VEGF-D. Another coreceptor known as neuropilin (NP) also modulates receptor ligand interactions of the VEGF family. NP-1 enhances the VEGF-A_165_ binding with VEGFR-2 whereas NP-2 acts as a functional receptor for VEGF-A_145_ and VEGF-A_165_. VEGF is expressed and secreted by majority of neuroblastoma cell lines and primary tumors that contribute to the growth of endothelial cells *in vitro* and angiogenesis *in vivo* leading to poor prognosis in high-risk neuroblastoma [10]. VEGF-A_165_ mRNA expression is upregulated in stage III neuroblastoma [11]. The presence of VEGF, VEGFR-1, and VEGFR-2 mRNA expression is evident in neuroblastoma surgical specimens and cell lines by reverse transcriptase-polymerase chain reaction (RT-PCR), but neuroblastoma SK-N-BE cell line expresses only the mRNA of VEGFRs [12]. Hypoxia upregulates VEGF expression and in conjugation with Flt-1 plays a pivotal role in VEGF-mediated autocrine signaling of tumor growth and angiogenesis in neuroblastoma cell line. A hypoxia-driven VEGF/Flt-1 autocrine loop acts together with hypoxia inducible factor-1 alpha (HIF-1*α*) through a mitogen-activated protein kinase and extracellular signal-regulated kinase-1/2-mediated pathway in neuroblastoma SK-N-BE2 cell line resulting in tumor cell survivability, multiple drug resistance, and neuroblastoma vascularity [13]. Neuroblastoma cells utilize VEGF both as a stimulator of angiogenesis and an inhibitor of apoptosis through upregulation of Bcl-2 expression [14]. Treatment of neuroblastoma in both *in vitro* and *in vivo* models with anti-VEGF agent results in decrease in tumor vascularity. In both neuroblastoma cell line and *in vivo* models, treatment with bortezomib, a proteasome inhibitor, showed anti-tumor activity with marked decrease in intratumoral vessel counts and reduction in VEGF expression suggesting diversified role of VEGF in progression of advanced stage neuroblastoma [15, 16]. The anti-VEGF antibody bevacizumab markedly reduced the growth rate of three malignant neuroblastoma SK-N-AS, IMR-32, and SH-SY5Y xenografts in immunodeficient mice [17]. ZD6474, a dual tyrosine kinase inhibitor of VEGFR-2 and EGFR, inhibited the phosphorylation of receptor tyrosine kinase in neuroblastoma cells leading to an increase in endothelial cell apoptosis and showed significant anti-tumor activity in seven neuroblastoma cell lines (SK-N-SH, SK-N-SH, SK-NAS, NGP, CHP-134, SH-SY5Y, and SH-EP) [18]. Though VEGF is indispensable for neuroblastoma angiogenesis, treatment of neuroblastoma xenograft model with anti-VEGF antibody shows decrease in vascularity and only partial regression of tumor, suggesting that VEGF blockade is not sufficient to prevent neuroblastoma angiogenesis or apoptosis and multiple angiogenic factors are needed for progression of neuroblastoma [19]. High levels of expression of VEGF-A_121_, VEGF-A_165_, VEGF-B, VEGF-C, FGF-2, Ang-2, TGF-*α*, and PDGF-A have been documented in an analysis of 22 neuroblastoma cell lines and 37 tumor samples suggesting that multiple angiogenic growth factors interplay in the regulation of neovascularisation in advanced stage neuroblastoma [20]. Expression of mRNAs in 24 neuroblastoma cell lines and 40 tumor samples using RT-PCR suggests that expression of c-Kit, PDGFR-*β*, and Flt-3 mRNA is associated with neuroblastoma in patients under 1 year, while the loss of expression of these kinases is associated with N-Myc amplification in patients over 1 year of age with advanced stage of neuroblastoma [21].

#### 3.1.2. PDGF and PDGFR Family

PDGF and their cognate receptor tyrosine kinases have potent implication in modulating endothelial cell proliferation and angiogenesis in solid tumors. Neuroblastoma cell lines also express dimeric isoforms of PDGF: PDGF-AA and PDGF-BB and their functional receptors PDGFR-*α* and PDGFR-*β*, respectively. PDGF has trophic effects on dopaminergic neurons *in vitro*. NB41, a mouse neuroblastoma cell line produces PDGF-AA, PDGF-BB and PDGFR-*β*, responding to PDGF-BB but not to PDGF-AA. Introduction of an antisense PDGFR-*β* RNA in NB41 cells completely suppressed neurite extension and cell growth indicating proliferative activity of PDGF in tumors [22]. Treatment of 7 neuroblastoma cell lines with imatinib mesylate displayed concentration-dependent decreases in cell viability and induction of apoptosis due to suppression of c-Kit and PDGFR phosphorylation, leading to inhibition of growth of neuroblastoma [10]. Treatment of 3 neuroblastoma cell lines such as SK-N-AS, IMR-32, and SH-SY5Y with SU11657, which is a multiple inhibitor targeting PDGFR, VEGFR, and c-Kit, reduced their expression and tumor angiogenesis by 63–96% also signifying the crucial roles of these angiogenic ligands and their receptors in tumor cell proliferation and survival [23]. PDGF mainly induces its activity through Ras activation followed by MAPK mediated action. Treatment of neuroblastoma SH-SY5Y cell line with somatostatin causes anti-proliferative effect by inhibition of PDGF-induced PDGFR phosphorylation followed by Ras inactivation providing multiple regulatory role of PDGF in cell proliferation and angiogenesis in high risk neuroblastoma [24].

#### 3.1.3. Stem Cell Factor and c-Kit Receptor Family

Produced by marrow stromal cells, stem cell factor (SCF) is a glycoprotein hemopoietin growth factor that acts by binding to its specific surface protein receptor encoded by the *c-Kit* proto-oncogene. SCF and c-Kit mRNAs are expressed in neuroblastoma cell lines and tumors, and they regulate tumor growth, survivability, and angiogenesis [25]. Neuroblastoma cells treated with an antibody to c-Kit augment apoptosis [26]. Both SCF and c-Kit are preferentially expressed in N-Myc amplified neuroblastoma tumors, and their signaling is active in promoting neuroblastoma cell proliferation that can be selectively inhibited by treatment with STI-571, a tyrosine kinase inhibitor [27]. Retinoic acid treatment has been shown to induce neuronal differentiation as well as enhanced SCF production in neuroblastoma cell lines [28]. Anti-c-Kit administration in 5 neuroblastoma cell lines IMR-5, SK-N-SH, SK-N-BE, AF8, and SJ-N-KP and the neuroepithelioma (NE) line CHP-100 showed significant decrease in cell viability due to induction of apoptosis suggesting that SCF is produced by some neuroblastoma cell lines via an autocrine loop to protect them from apoptosis and stimulate neuroblastoma growth and metastasis [29].

#### 3.1.4. Flt-3 and Receptor Family

Flt-3 is a transmembrane glycoprotein receptor structurally related to macrophage colony stimulating factor receptor-1 (CSF-1) and c-Kit, and it is expressed on several cell types including neuroblastoma cells. Flt-3 ligand (FL) is a cytokine that promotes the survival, proliferation, and differentiation of hematopoietic progenitors in synergy with other growth factors such as SCF, IL-3, IL-6, IL-12, and GM-CSF. The levels of expression of Flt-3 and FL in 12 tumor cell lines from neuroectodermal tumor (NET), Ewing's sarcoma (ES), and peripheral neuroectodermal tumor (PNET) and in 38 biopsies were analyzed [26]. RT-PCR and flow cytometry confirmed the presence of membrane and cytoplasmic Flt-3 and membrane FL in all these lines. FL shows a significant proliferating and anti-apoptotic activity in neuroblastoma and neuroepithelioma lines suggesting that Flt-3 and its ligand are expressed in neural crest-derived tumors and promote survival and proliferation of their cell lines [26].

#### 3.1.5. MMP Expression in Human Neuroblastoma

In advanced stages of neuroblastoma, tumor cell secrets MMP favoring degradation of extracellular matrix and enhancing tumor dissemination. The family of MMPs includes 72 kDa MMP-2 (or gelatinase A) and 92 kDa MMP-9 (or gelatinase B) that are collagenases. MMP-2 and MMP-9 facilitate invasion and metastasis, and they degrade important constituents of the interstitial stroma and subendothelial basement membrane type IV, V, VII, and X collagens and fibronectin [14]. Immunohistochemical analysis of the expression patterns of MMP-2, MMP-9, and their specific inhibitor, namely, tissue inhibitor of matrix metalloproteinase-2 (TIMP-2) in 31 neuroblastoma patients revealed that increased expression of MMP-2 but not that of MMP-9 and decreased expression of TIMP-2 in stromal tissues of neuroblastoma had significant association with progression of advanced stage disease [30]. Stromal MMP-9 regulates the vascular architecture in neuroblastoma by promoting pericyte recruitment. Prinomastat, a synthetic inhibitor of MMPs, shows inhibition of tumor cell proliferation in human neuroblastoma SK-N-BE2 orthotopically xenotransplanted tumors in immunodeficient mice and prolonged survivability, suggesting that advanced stages of neuroblastoma show increased expression of both MMP-2 and MMP-9 [31]. PEGylated SN38 (EZN-2208), a novel topoisomerase I inhibitor, showed promising anti-neuroblastoma efficacy through increasing apoptosis and reducing expression of VEGF, MMP-2, and MMP-9 in preclinical *in vitro* and *in vivo* models of human neuroblastoma [32]. MMP-2 remains in an inactive form in tumor cell lines and tumor tissues in the absence of expression of a membrane-type 1-MMP (MT1-MMP), which converts pro-MMP-2 to its activate form. MMP-9 is not expressed in neuroblastoma cell lines, but it is present in both inactive and active forms in tumor tissues [33]. Overexpression of MT1-MMP is highly associated with the advanced stage high-risk neuroblastoma. Increased expression of both MMP-2 and MMP-9 is also evident from studies in two neuroblastoma cell lines (LAN-5 and GL-LI-N) and immunohistochemical analysis of tissue biopsies of human neuroblastoma indicating that expression of these MMPs is correlated with angiogenesis in advanced stages [14]. MMPs have a role in retinoic acid-induced differentiation in neuroblastoma cell lines. For example, retinoic acid treatment for 24 h transiently increased invasion and expression of MMP-9 in SH-SY5Y and LAN-5 cells and MMP-2 in SMS-KCNR cells. MMP inhibition prevented retinoic acid-induced neurite formation indicating a regulatory role of MMP in differentiation [34]. Human neuroblastoma tumors xenotransplanted in MMP-9 (+/+) and MMP-9 (−/−) mice showed that bone marrow-derived MMP-9 regulates the recruitment of leukocytes and endothelial cells along with pericytes from bone marrow into tumor stoma leading to neovascularization and tumor progression [35]. N-Myc and Bcl-2 co-expression induces MMP-2 secretion and activation leading to tumorigenic phenotype in human neuroblastoma cells [36].

#### 3.1.6. Association of N-Myc Amplification with Human Neuroblastoma

*N-Myc*, a proto-oncogene normally expressed in the developing nervous system, is frequently overexpressed in high-risk neuroblastoma manifesting increased vasculature and poor prognosis. Amplified N-Myc down regulates both the production and activity of angiogenesis inhibitors and also provokes tumor malignancy [37]. N-Myc contains an N-terminal transactivation domain (Myc box) and a C-terminal basic helix-loop-helix/leucine zipper (bHLH-LZ) motif. The bHLH-LZ region is responsible for both DNA binding and interactions with other bHLH-LZ proteins (Max and Mad). 

N-Myc is normally located on the distal short arm of chromosome 2 (2p24), but in cells with N-Myc amplification it also maps to the double minute chromatin bodies (DM) or homogenously staining regions. Genetic material from chromosome 2p24 is amplified to form an extrachromosomal circular element or is transposed to the DM with retention of the normal copies of N-Myc at 2p24. Amplification of the N-Myc occurs in 20% to 25% of neuroblastomas and is a marker of aggressive tumor phenotype [38]. 

**Figure 2:**
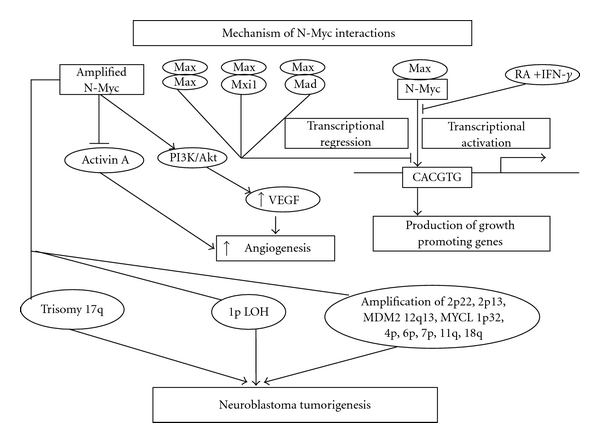
Schematic presentation of molecular changes associated with N-Myc amplification in neuroblastoma. Amplified N-Myc after heterodimerization with Max augments synthesis of several genes and through PI3K/Akt-mediated pathway regulates VEGF signaling resulting in neuroblastoma tumorigenesis.

After genomic amplification, N-Myc acts as transcription activator by heterodimerization with Max, a ubiquitously expressed nuclear protein lacking N-terminal transactivating domain. Interaction of the N-Myc/Max complex with the promoter region of target genes through the DNA sequence E-box motif (CACGTG) leads to progression of the cell cycle through the G1 phase ensuing increased transcription of several genes like *ODC*, *MCM7*, and *MRPI*. In the absence of N-Myc, other nuclear proteins Mad and Mxi1 competitively bind with Max and act as transcriptional repressor protein complex to inhibit transcription (Figure 2). Treatment of the N-Myc-amplified neuroblastoma cells with combination of IFN-*γ* and retinoic acid down regulates N-Myc expression through increased protein turnover, upregulates Mad1 mRNA and protein, and reduces N-Myc/Max heterodimerization, and predominates Mad1/Max network resulting in repression of the N-Myc target genes and potentiating differentiation and growth inhibition in neuroblastoma cells [39] (Figure 2). 

Overexpression of N-Myc down regulates the production of the anti-angiogenic factor activin A (Figure 2). Increased activin A expression inhibits neuroblastoma growth and angiogenesis in a neuroblastoma xenograft model, shows anti-proliferative activity, decreases colony formation of human neuroblastoma cell lines with amplified N-Myc, and induces differentiation. Amplified N-Myc through its interaction with the activin A promoter suppresses activin A synthesis resulting in enhanced vascularization to allow neuroblastoma progression [37]. 

Other genetic changes associated with N-Myc amplification in high-risk neuroblastoma are loss of heterozygosity on chromosome 1p and amplification of DNA on 2p22, 2p13, the *MDM2 *gene on 12q13, and the *MYCL *gene on 1p32. Comparative genomic hybridization reveals that amplifications of 4p, 6p, 7q, 11q, and 18q concurrently with N-Myc amplification have occurrence in advanced stage neuroblastoma. Allelotyping and comparative genomic hybridization studies indicate that trisomy for the long arm of chromosome 17 (17q) along with amplified N-Myc is indicative marker of aggressive neuroblastoma. Deletion of 11q is a predictive pointer in clinically high-risk neuroblastoma patients without N-Myc amplification [38] (Figure 2). N-Myc-amplified neuroblastoma cell lines either do not express CD44 or express a non-functional receptor. Absence of functional CD44 hyaluronan receptor on human N-Myc-amplified neuroblastoma cells predicts risk of disease progression and dissemination [40]. 

N-Myc plays an important role in the phosphatidylinositol-3-kinase- (PI3K-) mediated VEGF regulation in neuroblastoma cells as evident from the study where inhibition of N-Myc expression by siRNA transfection significantly blocks VEGF secretion [41]. Targeted inhibition of N-Myc by peptide nucleic acid (PNA) in human neuroblastoma N-Myc-amplified (GI-LI-N) and N-Myc-unamplified (GI-CA-N) cells showed cell cycle inhibition with induction of neuronal differentiation and apoptosis in N-Myc amplified cells and caused significant reduction in cell viability with N-Myc translation inhibition, accumulation of cells in G1, and induction of differentiation and apoptosis [42]. N-Myc silencing induces differentiation and apoptosis in human neuroblastoma cells. Also, siRNA directed to N-Myc may provide a novel therapeutic approach for an effective treatment of aggressive neuroblastomas [43].

#### 3.1.7. Neurotrophin Signaling Pathways in Human Neuroblastoma

Malignant transformation of sympathetic neuroblasts to neuroblastoma cells is regulated by neurotrophin receptor pathways. The tropomyosin related kinase (Trk) family consists of three receptor tyrosine kinases (TrkA, TrkB, and TrkC) each of which can be activated by one or more of the 4 neurotrophins such as nerve growth factor (NGF), brain-derived neurotrophic factor (BDNF), neurotrophin 3 (NT3), and neurotrophin 4 (NT4). Neurotrophins through intracellular signal transduction pathways mediate diversified biological activities such as cell survival, proliferation, and differentiation in normal and neoplastic neuronal cells. High expression of TrkA is present in neuroblastomas with favorable prognosis correlating with patient survival and absence of N-Myc amplification. Binding of transmembrane homodimer receptor TrkA to a homodimer of NGF leads to autophosphorylation of the receptor, docking of signaling proteins, signal transduction, and induction of gene transcription. TrkA through direct interaction with proteins SHC, PLC*γ*1, SH2B, IAPs, and Ras/MAPK signaling pathway regulates survival and differentiation and via activation of PI3K/Akt pathway mediates an alternative survival signaling pathway. TrkB is mainly expressed on unfavorable, aggressive neuroblastomas, responsible for both enhanced angiogenesis and drug resistance and strongly associated with N-Myc amplified tumors. Biological effects of TrkA and TrkB expression on neuroblastoma angiogenesis were examined in human neuroblastoma SH-SY5Y cell line and its TrkA and TrkB transfectants. TrkA expression inhibits tumor growth and down regulates the angiogenic factors VEGF and FGF-2 in SH-SY5Y cells whereas TrkB transfectants and parental SH-SY5Y cells induced endothelial cell proliferation and migration [44]. TrkA expression resulted in severely impaired tumorigenicity and invasiveness in SH-SY5Y xenografted mice and was associated with reduced angiogenic factor expression and vascularization of tumors [44]. NT expression pattern and mutational events leading to tumorigenesis divided neuroblastomas in distinct three types [38]. The first type is characterized by TrkA expressing tumors having mitotic dysfunction leading to near triploid (3N) karyotype. They can differentiate in response to NGF or undergo apoptosis in absence of NGF. Second type is characterized by TrkB expressing tumors having near diploid (2N) or tetraploid karyotype with genomic instability (gain of distal 17q material, loss of 11 q and/or 14 q material with 17q gain, and without 1p deletion and N-Myc amplification) and chromosomal structure alterations. Third type is characterized by tumors with 1p loss with N-Myc amplification [38]. Expression of different members of Trk family thus plays a key role in heterogenous clinical outcome of neuroblastoma.

#### 3.1.8. Expression of Vascular Integrins Associated with Human Neuroblastoma

Angiogenic neovascularization is also influenced by selective expression of adhesion receptor integrins. Cell adhesion to the extracellular matrix is mediated by integrins, which are heterodimeric transmembrane proteins comprising of a diverse family with over 15*α* and 8*β* subunits. Integrins *α*_v_ *β*_3_ and *α*_*v*_ *β*_5_ are expressed in high-risk neuroblastoma [45]. The binding of the integrin *α*_*v*_ *β*_3_ to its receptor provides a signal that causes reduction in p53, p21^Waf1^, and Bax expression and increase in Bcl-2 expression implicating survival of endothelial cells [46]. Expression of the integrins *α*_*v*_ *β*_3_ and *α*_*v*_ *β*_5_ by microvascular endothelium was demonstrated by immunohistochemical analysis [45]. The integrin *α*_*v*_ *β*_3_ was expressed by 61% of microvessels in high-risk neuroblastomas (stage IV and N-Myc-amplified stage III) and 18% of microvessels in low-risk tumors (stages I and II and non-N-Myc amplified stage III). The integrin *α*_*v*_ *β*_5_ was produced by 60% of microvessels in stage IV tumors suggesting their association with neuroblastoma aggressiveness [45]. *In vivo* echographic evidence of tumoral vascularization and microenvironment interactions in metastatic orthotopic human neuroblastoma xenografts showed highly angiogenic integrin *α*_*v*_ *β*_3_  marker indicating neovascularization to promote the role of cell adhesion molecules [47].

#### 3.1.9. Osteopontin in Neuroblastoma

Osteopontin (OPN) is a multifunctional phosphoprotein secreted by multiple cell types including osteoclasts, lymphocytes, and macrophages, and OPN is expressed in various tumor cells to act as a potent angiogenic factor contributing to tumor growth. OPN through its interaction with *α*_*v*_ integrins regulates both cell attachment and cell signaling. OPN in association with VEGF stimulates endothelial cell migration [48]. OPN plays an important role in increasing tumorigenicity through the enhancement of angiogenesis *in vivo*. Culture medium with murine neuroblastoma C1300 cells transfected with OPN gene significantly stimulates human umbilical vein endothelial cell migration and induces neovascularization in mice resulting in significant increase in tumor growth [49]. Presence of FGF-2 stimulates OPN upregulation in endothelial cells resulting in OPN-mediated recruitment of pro-angiogenic monocytes, induction of expression of the angiogenic cytokines TNF-*α* and IL-8 to contribute to amplification of FGF-2-induced neovascularization during inflammation, wound healing, and tumor growth [50].

#### 3.1.10. Role of HGF/c-Met Signaling in Neuroblastoma

Both N-Myc amplification and elevated expression of the neurotrophin receptor TrkB are correlated with the malignant phenotype of neuroblastoma. The TrkB-mediated invasiveness is associated with upregulation of hepatocyte growth factor (HGF) and its receptor c-Met [51]. HGF and c-Met are heterodimers composed of a *α*-chain subunit and a *β*-chain subunit linked by a disulfide bond. Mature HGF is a heterodimer composed of a *α*-chain (69 kDa), which contains an N-terminal hairpin domain and four kringle domains, and a *β*-chain (34 kDa) having serine-protease-like domain. Mature c-Met is comprised of a glycosylated *α*-subunit (50 kDa) and a transmembrane *β*-subunit (145 kDa). The extracellular region of mature c-Met contains a Sema domain, which is a cysteine-rich Met-related sequence (MRS) domain, and four immunoglobulin-like structure domain. The intracellular region is composed of a juxtamembrane domain, which is a tyrosine kinase domain (Tyr 1234 and Tyr 1235) that regulates kinase activity of c-Met, and a C-terminal regulatory tail (Tyr 1349 and Tyr1356) that is responsible for c-Met-regulated signal transduction pathway [52].

After binding of HGF to c-Met or other receptor tyrosine kinases, the HGF/c-Met signaling is initiated. Dimerization of c-Met causes transphosphorylation of tyrosines (Tyr 1234 and Tyr 1235) in the kinase domain followed by additional phosphorylation of other tyrosines (Tyr 1349 and Tyr 1356) in the C-terminal regulatory tail. c-Met transmits HGF signaling in cells via various downstream effectors such as Src/FAK, which regulates cell migration and adhesion. The other HGF/c-Met-associated effectors such as p120/STAT3 pathway augments neovasculature of cells whereas the PI3K/Akt and Ras/MEK pathways are responsible for cell proliferation and cell survival [52]. Thus, HGF mediates these multiple signaling cascades in endothelial cells directly or via involvement of VEGF and its receptor to promote angiogenesis. The important downstream effector of HGF/c-Met signaling that promotes neuroblastoma progression is STAT3 [53], one of the well-recognized signal transduction pathways involved in angiogenesis and other steps in cancers.

## 4. Anti-Angiogenic Therapy for Controlling Neuroblastoma

### 4.1. Single Anti-Angiogenic Agent Therapy for Controlling Neuroblastoma

#### 4.1.1. Inhibition of VEGF and VEGFR

VEGF is a critical mitogen regulating growth, neovascularization, and migration of endothelial cells and is associated with poor prognosis in neuroblastoma. VEGF and its receptors are expressed in human neuroblastoma tumors and cell lines. VEGF acts primarily by binding to one of its cognate receptors (VEGFR-1, VEGFR-2, and VEGFR-3) on endothelial cells, leading to autophosphorylation of tyrosine residues followed by subsequent activation of multiple intracellular signal transduction pathways such as the MAPK and PI3K/Akt pathways. Several experimental therapeutic strategies including VEGF inhibitors, antibodies directed against VEGF or against its receptors, soluble truncated receptors, introduction of anti-sense VEGF RNA, development of dominant-negative VEGF mutants and agents interfering directly with VEGF signal transduction have been emerged to target the interaction of VEGF with its receptors and thereby to suppress growth of neuroblastoma [54]. 

AZD2171, a selective inhibitor of the VEGF receptor family, displayed inhibition of tumor growth in neuroblastoma xenografts showing a promising anti-angiogenic treatment strategy for solid tumors [55]. Treatments with SB202190 (the p38 MAPK inhibitor) enhanced VEGF-mediated protection of the serum-deprived neuroblastoma SK-N-SH cells by reducing caspase-3 and caspase-7 activities and increasing the phosphorylation of the extracellular signal-regulated kinase 1/2 (ERK1/2) and Akt signaling pathway through activation of VEGFR-2. A blockade of VEGFR-2 signaling with the selective inhibitor SU1498 or gene silencing with VEGFR-2 siRNA in SB202190 treated cells hindered this prosurvival response and highly induced activation of caspase-3 and caspase-7, indicating that p38 MAPK exerts a negative effect on VEGF-mediated signaling through VEGFR-2 in serum-starved neuroblastoma cells [56]. Combinatorial treatment with vinblastine, a monoclonal antibody (DC101) targeting VEGFR-2 and rapamycin (mTOR inhibitor) in both neuroblastoma cells and orthotopic xenografted mice showed significant inhibition of tumor growth, angiogenesis, and reduction in microvessel formation suggesting that this combination may be relevant to design new curative strategies against neuroblastoma [57]. Argatroban (a derivative of arginine and a potent anti-coagulant and anti-thrombin agent) serves as a useful therapeutic tool for inhibition of thrombin-induced VEGF production in human neuroblastoma (NB-1) cells, and it may be effective in controlling disorders linked to thrombin-induced VEGF production in neuronal cells [58]. 

Inhibitors of VEGF with different specificities have been evaluated in human neuroblastoma NGP-GFP xenografts in nude mice [19]. These anti-VEGF agents are NX1838 (an antihuman VEGF_165_ RNA-based fluoropyrimidine aptamer), monoclonal anti-human VEGF antibody, and VEGF-Trap (a soluble composite decoy receptor consists of Ig-like domains of VEGFR-1 and VEGFR-2). High-dose VEGF-Trap showed the greatest inhibition of tumor growth leading to regression of tumor vasculature in xenograft model of neuroblastoma [19]. Combination of topotecan (a topoisomerase I inhibitor) with anti-VEGF antibody significantly suppressed tumor growth in neuroblastoma xenograft [59]. Continuous low-dose therapy with vinblastine and VEGFR-2 antibody induces sustained tumor regression in neuroblastoma xenograft, diminishes tumor vascularity, and directs inhibition of angiogenesis [60], suggesting that metronomic therapy can inhibit endothelial cell proliferation, angiogenesis, and tumor growth.

#### 4.1.2. Inhibition of Multiple Angiogenic-Related Factors

VEGF, FGF-2, PDGF, SCF, and their cognate receptor tyrosine kinases are strongly implicated in angiogenesis in solid tumors like neuroblastoma. SU11657 (SUGEN) is a selective multitargeted (class III/V) tyrosine kinase inhibitor with anti-tumor and anti-angiogenic activity exerted by targeting PDGFR, VEGFR, SCF receptor (c-Kit), and FMS-related tyrosine kinase 3. Oral administration of SU11657 caused significant inhibition of tumor growth as well as of angiogenesis in SK-N-AS, N-Myc amplified IMR-32, and SH-SY5Y human neuroblastoma xenografted athymic nude mice. Immunohistochemical analysis revealed down regulation of the expression of VEGFR-2, PDGFR-*β*, and c-Kit suggesting that targeting of class III/V receptor tyrosine kinases and their ligands can suppress neuroblastoma tumorigenicity and angiogenesis [23]. Imatinib mesylate, a selective inhibitor of the tyrosine kinase c-Kit and PDGFR, displayed concentration-dependent decreases in cell viability, induction of apoptosis with ligand-stimulated phosphorylation of c-Kit and PDGFR, and inhibition of VEGF expression in 7 neuroblastoma cell lines. Oral imatinib therapy showed anti-tumor efficacy in xenografted SCID mice [10]. SU6668, another receptor tyrosine kinase inhibitor of VEGFR2, PDGFR, and FGFR1, in combination with cyclophosphamide significantly inhibited VEGFR-2 and tumor growth in human neuroblastoma xenografts [37]. Therefore, therapy targeted to multiple angiogenic factors appears to be a novel treatment modality in neuroblastoma.

#### 4.1.3. Retinoids for Inhibition of Angiogenesis in Neuroblastoma

**Figure 3:**
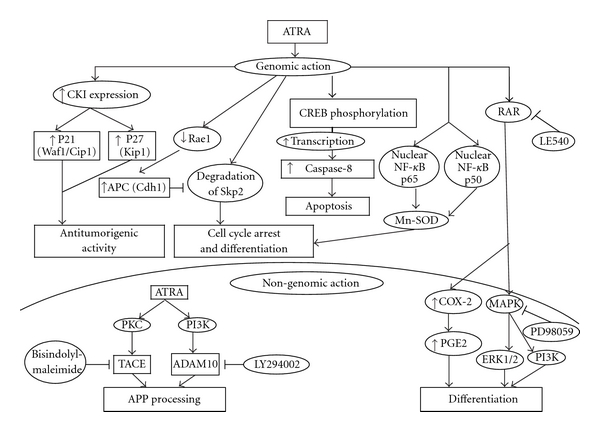
Genomic and non-genomic actions of all-*trans* retinoic acid (ATRA). Genomic action of ATRA includes caspase-8 transcription, cell cycle arrest, and apoptosis. Non-genomic action of ATRA involves induction of PI3K/Akt pathway for upregulation of secretase. ATRA activates retinoic acid receptor (RAR) and MAPK signaling pathway for contribution to differentiation in neuroblastoma.

Retinoids, a class of natural or synthetic compounds structurally related to vitamin A, hold great promises for the prevention and treatment of cancer. The cell differentiating properties and anti-cancer activity of the retinoids all-*trans* retinoic acid (ATRA) and 13-*cis* retinoic acid (13-CRA) are well established in several *in vitro* and *in vivo* models [61, 62]. Retinoids are signaling molecules that are involved in proliferation, differentiation, and apoptosis both via non-receptor and nuclear-receptor-mediated pathways thereby altering gene expression. ATRA induces the expression of both mRNA and protein of the differentiation marker manganese superoxide dismutase (MnSOD) in human neuroblastoma (SK-N-SH) cells with involvement of NF-*κ*B and SOD2 genes, contributing to the concept of using retinoids in cancer therapy [63]. Retinoic acid reduces human neuroblastoma cell migration and invasiveness as evident from down regulation of expression of doublecortin (a microtubule-associated protein involved in neuronal migration) and lissencephaly-1 (another protein involved in neuronal migration) and upregulation of expression of neurofilament protein-68 in human neuroblastoma SK-N-SH cell line [64]. Retinoic acid causes PI3K and PKC-dependent upregulation of 2 putative *α*-secretases and the disintegrin metalloproteinases ADAM10 and TACE, stimulates *α*-secretase processing of amyloid precursor protein (APP), and down regulates *β*-secretase cleavage thereby leading to suppression of amyloid-*β* formation in human neuroblastoma SH-SY5Y cells (Figure 3). The PI3K inhibitor LY294002 and the PKC inhibitor bisindolylmaleimide XI reduced the retinoid-mediated effect on ADAM10 protein levels and completely abolished the effect on TACE indicating involvement of PI3K- and PKC-mediated signaling pathway in retinoic acid-induced upregulation of secretase [65]. Combined IFN-*γ* and retinoic acid treatment targets the N-Myc/Max/Mad1 signaling pathway and represses expression of the N-Myc/Mad1 target genes ornithine decarboxylase and hTERT, indicating that this combination strategy may have therapeutic benefits in targeting N-Myc function in high-risk, N-Myc-amplified neuroblastoma patients [39]. Non-genomic actions of retinoic acid on neuroblastoma SH-SY5Y cells is mediated by the classical nuclear receptor, retinoic acid receptor (RAR), resulting in activation of PI3K and MAPK signaling pathways contributing to retinoic acid-induced differentiation [66]. Combination of retinoic acid with histone deacetylase inhibitors (HDACi) could result in improved anti-tumorigenic activity. The HDACi trichostatin A (TSA), sodium butyrate, and suberoylanilide hydroxamic acid (SAHA) alone and in combination with retinoic acid can increase cyclin kinase inhibitor (CKI) mRNA levels in human neuroblastoma SH-SY5Y cells, and activate CKI promoters to inhibit tumor cell growth in neuroblastoma [67]. ATRA through involvement and activation of RAR and ERK 1/2 induces COX-2 and prostaglandin E2 synthesis in human neuroblastoma SH-SY5Y cells and this effect is completely abolished by the RAR pan-antagonist LE540 or the MEK-1 inhibitor PD98059 suggesting involvement of RAR and kinase-dependent mechanisms for ATRA-induced COX-2 activity [68] (Figure 3). Genomic action of ATRA includes caspase-8 transcription via CREB-phosphorylation leading to apoptosis in neuroblastoma cells [69]. Retinoic acid induces cell cycle arrest and differentiation through degradation of the F-box protein Skp2 and stabilization of the cyclin-dependent kinase inhibitor p27 (Figure 3). Skp2 is degraded by anaphase-promoting complex (APC) (Cdh1). Retinoic acid downregulates Rae1 (a nuclear export factor), facilitates APC- (Cdh1-) mediated Skp2 degradation leading to the arrest of cell cycle progression and differentiation in neuroblastoma SH-SY5Y cells [70] (Figure 3). 

N-(4-hydroxyphenyl) retinamide (4-HPR), also known as fenretinide, is a synthetic retinoid that induces anti-proliferative activity and apoptosis, inhibits angiogenesis and cell motility, and decreases invasiveness in a wide variety of human cancer cell lines and mammary, prostate, and ovarian tumors in transgenic mice. 4-HPR can activate retinoid receptor-dependent and independent pathways for induction of apoptosis. 4-HPR induces the gene expression of BBC3, pro-apoptotic member of the Bcl-2 family, to trigger apoptosis in neuroblastoma cell lines [71]. 4-HPR also potentiates NF-*κ*B activity, I*κ*B*α* phosphorylation, production of reactive oxygen species (ROS), 12-lipoxygenase activity, and GADD153 transcription factor activity to elicit apoptotic response in neuroblastoma SH-SY5Y cells [72]. 4-HPR, IFN-*γ*, and demethylating agent 5-aza-cytidine activate the promoter region of caspase-8 in neuroblastoma cells and regulate both constitutive and inducible caspase-8 expression in pathophysiological condition [73]. 4-HPR is also found to upregulate ceramide level and metabolism of ceramide to gangliosides via glucosylceramide synthase and GD3 synthase leading to activation of ROS signaling pathway via 12-LOX resulting in oxidative stress and neuroblastoma cell death by induction of the transcription factor GADD153 and the Bcl-2-related Bak protein [74]. 4-HPR caused sustained activation of JNK/p38 MAPK pathway and augmented apoptosis in a ROS-dependent manner in neuroblastoma cells [75]. 4-HPR through inhibition of both VEGF and FGF-2 induced endothelial cell proliferation to downregulate angiogenesis in neuroblastoma [76]. Retinoid in combination with an anti-angiogenic factor may provide an effective treatment strategy for controlling neuroblastoma.

#### 4.1.4. Anti-Angiogenic Inhibitors of Methionine Aminopeptidase

The anti-angiogenic inhibitors of methionine aminopeptidase (MetAP2) may hold a key role in therapeutic management of neuroblastoma. MetAP2 is a cytoplasmic enzyme responsible for promoting endothelial cell proliferation, migration, and induction of angiogenesis in neuroblastoma [54]. The concentration of MetAP2 has been found to be higher in neuroblastomas [37]. 

TNP-470, an irreversible inhibitor of MetAP2, showed anti-angiogenic activity in preclinical models of neuroblastoma. TNP-470 as monotherapy or in combination with a cytotoxic agent such as cisplatin, paclitaxel, or cyclophosphamide significantly inhibited tumor angiogenesis [54]. TNP-470 significantly inhibited growth rate and tumorigenecity in several neuroblastoma xenografts [54, 77]. TNP-470 treatment in small neuroblastoma tumors was found to exhibit chromaffin differentiation and induction of apoptosis [54]. Microspheres containing TNP-470 strongly inhibit *in vivo* hepatic metastasis of neuroblastoma [78]. However, TNP-470 has a few limitations such as neurologic toxicities.

Another reversible inhibitor of MetAP2 is A-357300, which is a promising therapeutic agent against neuroblastoma. A-357300 alone or in combination with cyclophosphamide significantly caused tumor regression and increased survival rate in CHP-134-derived neuroblastoma xenografts without any toxicity [79]. A-357300 induces cell cycle arrest G1 phase in neuroblastoma cells, and showed anti-angiogenic and anti-tumor potential in neuroblastoma murine models [80]. A800141, another orally active inhibitor of MetAP2, showed potent anti-angiogenic and anti-tumor activities in several tumor xenografts including neuroblastoma, B-cell lymphoma, and colon and prostrate carcinomas [81].

#### 4.1.5. Endostatin for Inhibition of Angiogenesis in Neuroblastoma

Endostatin is a 20 kDa fragment of collagen XVIII that acts as an endogenous inhibitor of endothelial cell proliferation, tumor angiogenesis, and tumor growth. Recombinant human endostatin (rhEndostatin) worked as potent anti-angiogenic agent and was effective against human neuroblastoma xenograft model [82]. In another gene therapy approach, the angiogenesis inhibitor endostatin and the potent immunogen green fluorescent protein (GFP) were delivered to murine neuroblastoma cells prior to inoculation of tumor cells into syngeneic immunocompetent mice [54]. The combination of endostatin and GFP showed synergistic anti-tumor and immunogenic response suggesting that both anti-angiogenic and immunotherapeutic strategies worked to control neuroblastoma. Also, pre-existing primary neuroblastoma xenografts could hold back the growth of a new secondary subcutaneous tumor, inhibit angiogenesis, and induce apoptosis due to release of endostatin from the tumor [83].

#### 4.1.6. Targeting HGF/c-Met for Inhibition of Angiogenesis in Neuroblastoma

One promising strategy to inhibit angiogenesis in neuroblastoma is to target the signaling pathway of HGF (also known as scatter factor, SF) along with its receptor HGFR (also known as c-Met) that plays important roles in mitogenic, motogenic, and morphogenic regulation of angiogenesis, tumor growth, and metastasis. Inhibitors of this signaling pathway have been shown to inhibit angiogenesis in multiple *in vitro *and *in vivo *models of cancers and may be used as an effective therapy to treat cancers. HGF and c-Met are upregulated in many human cancers including neuroblastoma [84] and highly responsible for neuroblastoma invasion *in vitro *and *in vivo*. Therefore, inhibitors targeting HGF/c-Met signaling may be an effective therapeutic approach to control angiogenesis and prevent tumor growth. The first strategy for inhibition may be blocking the binding of HGF to c-Met with the use of HGF antagonists and antibodies against HGF/c-Met. The HGF/c-Met antagonists NK4, uncleaved HGF, Sema, decoy Met, and recombinant variant Met were used previously. The antibodies targeting HGF are L2G7 (Galaxy Biotech), AMG102 (Amgen), OA-5D5 (Genentech), and CE-3556221 (Pfizer) that can inhibit tumor angiogenesis by blocking the binding of HGF/c-Met [52]. In another study, it has been reported that inhibition of HGF activity by anti-HGF-antibodies or suppressing the function of c-Met by siRNA can repress the TrkB-induced invasiveness [51].

Next strategy is to inhibit the HGF/c-Met signaling pathway (a) by targeting Tyr kinase domain of c-Met for inhibiting tyrosine phosphorylation with inhibitors such as SU11274, PF2341066 (Pfizer), XL880, and XL184 (Exelixis) and (b) by targeting inhibitors of downstream effectors of HGF/c-Met signaling such as PI3K inhibitor LY294002 (to inhibit HGF/c-Met-induced cell motility) and MEK inhibitor PD98059 (to inhibit HGF/c-Met-mediated cell invasion). The other important inhibitors are Src inhibitor PD180970 and SU6656 that inhibit HGF/c-Met-induced Src and STAT3 activity [52]. Another inhibitor PHA665752 has been reported to suppress c-Met activity and block HGF-induced cell migration and proliferation of c-Met-positive neuroblastoma cells [85]. Thus, targeting HGF/c-Met signaling pathways could be a beneficial approach for controlling angiogenesis in neuroblastoma.

### 4.2. Combination Anti-Angiogenic Therapy to Treat Neuroblastoma

Despite multimodal myeloablative chemotherapy, this pediatric malignancy has a poor prognosis. Current therapeutic strategies including combination of novel targeted drugs such as signal transduction and angiogenesis inhibitor, differentiation and apoptosis inducers, and immunotherapeutic modulators can provide superior management and prevention of neuroblastoma. Combination of trichostatin A (TSA) and interferon-alpha (IFN-*α*) showed the most potent anti-angiogenic therapeutic strategy for devastating neuroblastoma [86]. Combination of TSA and IFN-*α* not only decreased endothelial cell migration, invasion, and capillary tubule formation but also inhibited expression of VEGF, HIF-1*α*, and MMP-9 in neuroblastoma cells [86]. Sunitinib (targeted against PDGFR and VEGFR) and rapamycin showed synergistic inhibition of tumor growth, angiogenesis, and anti-metastatic activity in both neuroblastoma cell culture and xenograft models suggesting the anti-cancer efficacy of this combination therapy [87]. The anti-VEGF antibody bevacizumab treatment in orthotopic neuroblastoma xenograft model causes normalization of the tumor vasculature due to VEGF blockade resulting in improved delivery and anti-tumor efficacy of chemotherapy [88]. Combination of the thrombospondin-1 peptide ABT-510 with valproic acid was highly effective to regress tumor growth and microvascular density in two different N-Myc-amplified cell lines-derived neuroblastoma xenografts when compared with monotherapy suggesting the potency of combination anti-angiogenic therapy for treatment of neuroblastoma [89]. Bortezomib (proteasome inhibitor) and 4-HPR showed synergistic anti-tumor and anti-angiogenic activities in neuroblastoma cell culture and xenograft models through involvement of endoplasmic reticulum stress response [90]. Anti-angiogenic agents (arginine deiminase, SU5416, and DC101) in combination with simultaneous irradiation inhibited *in vivo* growth of neuroblastomas with subsequent reduction in number of tumor vessels [91]. Valproic acid in combination with INF-*α* synergistically inhibited growth of malignant phenotype of human N-Myc-amplified neuroblastoma BE(2)-C cells [92]. This combination therapy caused marked downregulation of N-Myc, Bcl-2, and neural cell adhesion molecule with induction of differentiation in neuroblastoma [92].

### 4.3. Gene Therapy and Immunotherapy for Inhibition of Angiogenesis in Neuroblastoma

In gene therapy approach, genetic material encoding the therapeutic protein is transferred into the mammalian cells. Viral vector-mediated gene delivery of angiogenic inhibitors shows potent therapeutic promises against malignant neuroblastoma. Construction of retroviral and adenoviral vectors expressing the angiogenic inhibitor proteins is being tested in different neuroblastoma murine tumor models. In this approach, host cells are engineered to make the anti-angiogenic protein *in vivo* on long-term basis without daily administration of recombinant proteins. Single vector through the delivery of multiple genes can therefore target several angiogenic pathways. Gene therapy by retroviral vector for cell-mediated *in vivo *delivery of TIMP-3 could suppress tumor-induced angiogenesis and tumor growth [93]. Two replication-defective retroviral vectors were used to transduce murine neuroblastoma cells (NXS2). Single GFP expression was ineffective to reduce tumor growth whereas engineered expression of Flk-1 resulted in inhibition of endothelial cell proliferation and migration and also restriction of neuroblastoma growth [94]. Adeno-associated virus- (AAV-) mediated delivery of IFN-*β* with subsequent administration of TSA *in vitro* and *in vivo* in murine model of retroperitoneal neuroblastoma was highly effective, showing reduction in tumor cell count with elevated expression of p21 suggesting the potential of this therapy to treat this devastating disease [95]. AAV-mediated systemic delivery of human IFN-*β* (AAV-hIFN-*β*) in combination with low-dose cyclophosphamide showed anti-angiogenic activity with down regulation of VEGF and FGF-2 and also caused complete tumor regression in orthotopic retroperitoneal and disseminated models of neuroblastoma [95]. Triple combination of conditionally replicating oncolytic herpes simplex virus-1 vectors armed with IL-12, IL-18, or soluble B7-1 potentiated *in vivo* anti-tumor efficacy by augmenting T-cell-mediated response in neuroblastoma Neuro2a model [96]. Thus, insertion of immunostimulatory transgenes into viral genome could be a useful strategy for neuroblastoma therapy [96]. Neuroblastoma immunotherapy using cytokines may have direct anti-tumor and immunomodulatory effects. Co-transfection of IL-2 and IL-12 in Neuro2a cells down regulated *in vivo* tumorigenicity and provided CD4+ T cell and CD8+ T cell mediated immunotherapeutic responses in syngenic neuroblastoma model [97]. Immunogene therapy with IL-12- and IL-15-engineered neuroblastoma Neuro2a cells showed therapeutic efficacy by enhancement of CD8+ T-cell immunoresponses and potentiated the survivability of neuroblastoma-bearing syngenic mice [98]. Inhibition of angiogenesis by targeted immunotherapy may provide a novel treatment modality against neuroblastoma. Anti-angiogenic integrin *α*_*v*_ antagonist and antibody-IL-2 fusion protein induced tumor regression and dramatically decreased tumor vessel density in syngenic neuroblastoma model [99].

### 4.4. Vascular Targeting for Inhibition of Angiogenesis in Neuroblastoma

Vascular targeting has tremendous potential in neuroblastoma therapy. Vascular targeting with selective occlusion of pre-existing tumor blood vessels can induce tumor suppression by destruction of tumor vasculature and extensive necrosis. Two types of vascular targeting agents, small molecules (e.g., microtubule destabilizing agents, flavonoids), and ligand-based fusion proteins (e.g., immunotoxins, liposomally encapsulated drugs, antibodies conjugated with cytokines, and gene therapy) hold key role in tumor regression. Sterically stabilized immunoliposomal (SIL) therapy by targeted delivery of entrapped drugs to solid tumors can potentiate therapeutic efficacy. SIL loaded with doxorubicin (DXR) and targeted to the disialoganglioside receptor GD-2 in human neuroblastoma model can cause marked tumor regression. In neuroblastoma xenografts, the combined delivery of NGR peptide (targeting angiogenic endothelial cell marker aminopeptidase N) with DXR-loaded liposome showed dramatic tumor suppression by destruction of tumor vasculature [100]. Tumor vascular targeting by TNF-*α* with chemotherapeutic drug GNGRG peptide or coupling this peptide to the surface of liposomal DXR could augment the penetration of chemotherapeutic drugs in subcutaneous tumor models and therefore serve as a novel treatment strategy for neuroblastoma [101]. Immunotoxins- and antibody-based therapy showed promises in eradication of solid tumors. Cytokine gene transfection of tumor cells for induction of expression of major histocompatibility complex (MHC) class II in tumor endothelium was carried out in a neuroblastoma murine model [102]. The delivery of anti-class II-deglycosylated Ricin A chain caused destruction of IFN-*γ*-activated endothelial cells *in vitro* and complete thrombosis and decay of vasculature of neuroblastoma tumors *in viv*o suggesting that immunoconjugates attached with antibodies against tumor endothelium could offer extensive therapy to numerous solid tumors in humans [102].

## 5. Conclusion and Future Direction

Despite multimodal therapeutic approaches, the mortality rate is very high in patients with malignant neuroblastoma. Novel treatment strategies for improving patient survival and decreasing therapy-related toxicity are urgently warranted. Angiogenesis appears to be a primary requirement for growth, invasion, and metasatsis of malignant neuroblastomas. Inhibition of neovascularization by anti-angiogenic agent could provide promising therapeutic approach for treatment of neuroblastoma. Synergistic treatment modalities with the use of anti-angiogenic drugs in combination chemotherapy [103] and newer targeted therapy for inhibiting vascular sprouting and tumor vasculogenesis could provide effective tools for future therapies for this type of deadly cancer. Continuous low-dose chemotherapy or “metronomic dosing” could potentiate anti-angiogenic and apoptotic effects of cytotoxic agents on both proliferating endothelial cells and tumor cells. Metronomic scheduling of imatinib with the chemotherapeutic drug DXR showed anti-proliferative activity and apoptosis induction in both neuroblastoma cell lines and xenograft model [104]. Integrated treatment strategies for inducing tumor cell death, differentiation, and apoptosis, overcoming multiple drug resistance, and inhibiting angiogenesis with preclinical evaluation should provide justification for their future selection as clinical trials in high-risk neuroblastoma.
